# Epidemiology of intestinal parasitic infections in school children in Ghazni Province, eastern Afghanistan

**DOI:** 10.12669/pjms.316.8889

**Published:** 2015

**Authors:** Krzysztof Korzeniewski, Alina Augustynowicz, Agata Smoleń, Anna Lass

**Affiliations:** 1 Krzysztof Korzeniewski MD, PhD. Department of Epidemiology and Tropical Medicine, Military Institute of Medicine, Gdynia, Poland; 2 Alina Augustynowicz MSc. Department of Epidemiology and Tropical Medicine, Military Institute of Medicine, Gdynia, Poland; 3 Agata Smoleń MD, PhD. Department of Epidemiology, Medical University of Lublin, Poland; 4 Anna Lass PhD. Department of Tropical Parasitology, Medical University of Gdansk, Poland

**Keywords:** Intestinal parasites, Prevalence, Afghanistan

## Abstract

**Objective:**

To estimate the prevalence of intestinal parasites and their species in Afghan school children and to establish appropriate treatment methods for detected pathogens.

**Methods:**

Parasitological examination of stool samples collected from 1369 children aged 8-18, students of the Jahan Malika High School in Ghazni Province in eastern Afghanistan, was conducted in the period November 2013-April 2014. Three stool samples were collected from each patient every second day; the samples were fixed in 10% formalin and tested by light microscopy using the methods of direct smear in Lugol’s solution, decantation in distilled water, and Fülleborn’s flotation.

**Results:**

Of 535 examined children (39.1% of the study group) were infected with nematodes (n=324), cestodes (n=118), trematodes (n=12), and protozoa (n=228), 132 were diagnosed with co-infections (mainly ascariasis+giardiasis, ascariasis+hymenolepiasis) and received single or combined therapy.

**Conclusions:**

The Afghan community is an example of population characterized by a high rate of parasitic infections. Owing to high prevalence of multiple infections among inhabitants of Afghanistan, it seems that a mass deworming campaign with a single-dose chemotherapy may prove ineffective in eradicating intestinal parasites in the local population.

## INTRODUCTION

Intestinal parasitic infections belong to neglected diseases, one of the major health problems of the contemporary world.^1^ It is estimated, that over two billion people are infected with intestinal pathogenic parasites, and 5 billion live in areas where intestinal parasites are endemic.^2^ The incidence of gastrointestinal parasitic diseases is exceptionally high in developing countries, where soil and water contamination, a limited number of households with access to safe drinking water sources, a large number of asymptomatic carriers, low standards of hygiene, and lack of health care result in the spread of orally transmitted infections.^3^,^4^

Afghan community is an example of a population with estimated a high index of intestinal parasitic infections. According to the United Nations ranking, which classifies countries in terms of their wealth and economic development, Afghanistan is placed in the last positions.^5^ Afghans are dependent on international, humanitarian assistance. Owing to limited diagnostic capabilities of the national healthcare, reports on the prevalence of diseases in the general population of Afghanistan are rarely laboratory-confirmed. Any improvement in the health status of the Afghan population is additionally restrained by poor public awareness of the principles of disease prevention.^6^ According to the U.S. sources, the rates of amebiasis in the general population reach 3%, giardiasis has been found in nearly 11% of the examined children, and as much as 90% of the population may be infected with at least one intestinal parasite.^7^ The data, however, are only rough estimates based on the results of few screening studies.

The aim of this study was to estimate the prevalence of intestinal parasites and their species in the Afghan community on the example of school children living in eastern part of the country as well as to establish appropriate treatment methods for detected pathogens.

## METHODS

### Study population

In total, 1369 Afghan children aged 8-18, who were attending the Jahan Malika High School in Ghazni, capital city of Ghazni Province in eastern Afghanistan, were examined in this study. Parasitological examination was performed by medical personnel serving in ISAF military operation in Afghanistan, in cooperation with the Head of the Health Service Department in Ghazni Province Zia Ghul, MD and the Head of Ghazni Provincial Hospital (GPH) Baz Mohammat Hemmat, MD, in the period November 2013 - April 2014.

### Sample collection and laboratory procedures

Three stool samples were collected from each patient every second day, the samples were fixed in 10% formalin, and transported to the Military Institute of Medicine in Poland, where they were tested by light microscopy using the methods of direct smear in Lugol’s solution, decantation in distilled water, and Fülleborn’s flotation technique.^8^,^9^ Each of the samples collected from every patient was tested by means of all three methods. Thus, 12321 parasitological tests were performed in total.

### Ethical considerations

The research project was accepted by the Committee on Bioethics in the Military Institute of Medicine (Decision No 43/2014) on the basis of Declaration of Helsinki (1996) and the rules elaborated by the European Union “Good clinical practice for on medical products in the European Community. The rules governing medical products in the European Community” (1990) ratified by the Committee of Ethics in Poland (March 1993).

### Statistical analysis

The statistical analyses have been performed using the statistical suite StatSoft. Inc. (2011) STATISTICA (data analysis software system) version 10.0. www.statsoft.com (SN JGNP3087539302AR-E) and Excel. The quantitative variables were characterized by the arithmetic mean of standard deviation or median or max/min (range). The qualitative variables were presented with the use of count and percentage. Statistical significance of differences between two groups (unpaired variables model) was processed with the U Mann-Withney. Chi-squared tests for independence were used for qualitative variables (with the use of Yates correction for cell counts below 10, with check of Cochrane’s conditions or with Fisher’s exact test respectively). In all the calculations the statistical significance level of p=0.05 has been used.

## RESULTS

The mean age in the study group of 1369 Afghan children, students of the Jahan Malika High School in Ghazni, eastern Afghanistan, was 12.7 +/- 2.4 years (range 8-18, median 12.0). The mean age of females was 12.9 +/- 2.4 (range 8-18, median 12.0), and the mean age of males was 11.0 +/- 1.2 (range 8-14, median 11.0). The analysis of age and sex did not show statistically significant differences (U Mann-Whitney test 8.43, p<0.000001). The mean age of children without infections was 13.1 +/- 2.5 (range 8-18, median 13.0) and children with infections was 12.2 +/- 2.1 (range 8-18, median 12.0). The analysis of age and infection rates did not show statistically significant differences (U Mann-Whitney test 6.08, p<0.000001). There were 1257 (91.8%) females and 112 (8.2%) males in the study group. Infections were confirmed in 535 children (39.1% of all the study population), 499 females (93.7%) and 36 males (6.7%).

Parasitological examination, which revealed that 39.1% of the children attending the above-mentioned high school in eastern Afghanistan were infected with pathogenic intestinal parasites, helped implement appropriate treatment in line with current standards of the antiparasitic therapy.^7^ Of 535 children infected with nematodes (n=326, 23.8%), cestodes (n=118, 7.7%), trematodes (n=12, 0.9%), and protozoa (n=228, 16.7%), 132 were diagnosed with co-infections (mainly ascariasis+giardiasis, ascariasis+hymenolepiasis) and received combined therapy (albendazole+metronidazole, albendazole+praziquantel) (Table-I).

**Table-I:** Intestinal parasitic infections in students of the Jahan Malika High School in Ghazni, eastern Afghanistan, November 2013-April 2014.

Intestinal parasites	No. of infections (n=684)	Percentage of infected children (n=535)	Percentage of tested children (n=1369)	Treatment
Nematodes	326	60.9	23.8	
Ascaris lumbricoides	295	55.1	21.5	albendazole
Enterobius vermicularis	20	3.7	1.5	albendazole
Strongyloides stercoralis	3	0.6	0.2	albendazole
Ancylostoma duodenale/Necator americanus	8	1.5	0.6	albendazole
Cestodes	118	22.1	8.6	
Hymenolepis nana	89	16.6	6.5	praziquantel
Hymenolepis diminuta	13	2.4	0.9	praziquantel
Taenia spp.	16	3.0	1.2	praziquantel
Trematodes	12	2.2	0.9	
Dicrocoelium dendriticum	12	2.2	0.9	praziquantel
Protozoa	228	42.6	16.7	
Giardia intestinalis	224	41.9	16.3	metronidazole
Entamoeba histolytica s.l.	4	0.7	0.3	metronidazole
Number of infected children	535	100.0	39.1	

**Fig.1:**
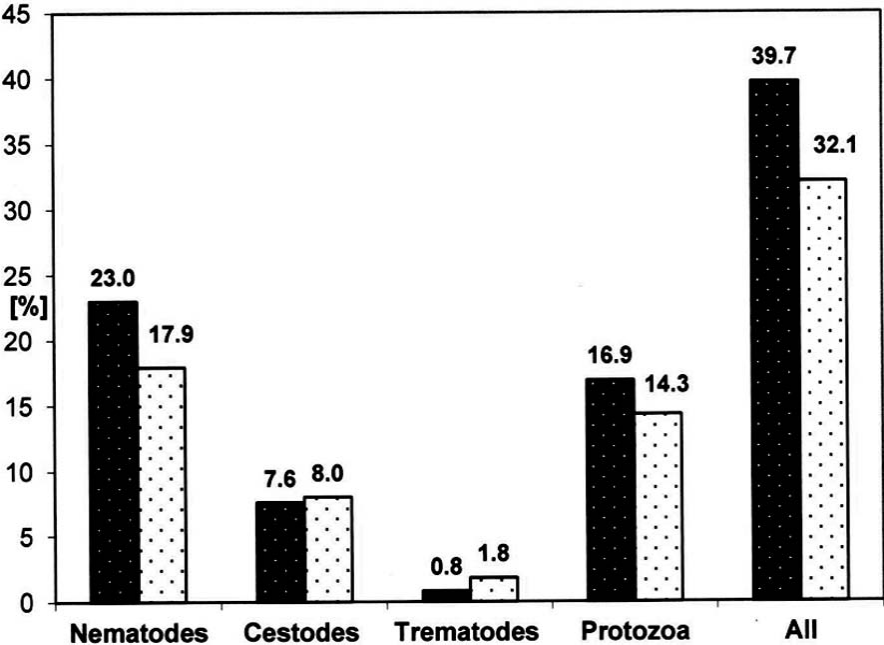
Distribution of parasitic infections in Afghan children by sex.

The analysis of the rates of infections with intestinal parasites among children by sex did not show any statistically significant differences (Table-II, Fig.1).

**Table-II:** Distribution of parasitic infections in Afghan children by sex.

Intestinal parasites	Females n=1257 (%)	Males n=112 (%)	p-value
Nematodes	304 (23.0)	22 (17.9)	0.21
Cestodes	109 (7.6)	9 (8.0)	0.88
Trematodes	10 (0.8)	2 (1.8)	0.58
Protozoa	212 (16.9)	16 (14.3)	0.57
Number of infections	635 (39.7)	49 (32.1)	0.12

Detailed information on the numbers and rates of intestinal parasites by age of the infected children is presented in Table-III and Table-IV.

**Table-III:** Distribution of parasitic infections among Afghan children by age.

Age	Nematodes n=326 (%)	Cestodes n=118 (%)	Trematodes n=12 (%)	Protozoa n=228 (%)	No. of infections n=684 (%)	No. of infected children n=535 (%)	No. of tested children n=1369 (%)
8	5 (1.5)	1 (0.8)	0 (0.0)	2 (0.9)	8 [1.2]	7 (1.3)	17 (1.2)
9	9 (2.8)	0 (0.0)	0 (0.0)	1 (0.4)	10 [1.5]	9 (1.7)	28 (2.0)
10	51 (15.6)	26 (22.0)	0 (0.0)	42 (18.4)	119 [17.4]	86 (16.1)	177 (12.9)
11	78 (23.9)	33 (28.0)	4 (33.3)	54 (23.7)	168 [24.6]	129 (24.1)	274 (20.0)
12	70 (21.5)	27 (22.9)	4 (33.3)	57 (25.0)	158 [23.2]	125 (23.4)	268 (19.6)
13	38 (11.7)	12 (10.2)	2 (16.7)	22 (9.6)	73 [10.7]	56 (10.5)	155 (11.3)
14	27 (8.3)	9 (7.6)	0 (0.0)	15 (6.6)	51 [7.5]	44 (8.2)	121 (8.8)
15	18 (5.5)	8 (6.8)	1 (8.3)	14 (6.1)	41 [6.0]	33 (6.2)	129 (9.4)
16	18 (5.5)	2 (1.7)	1 (8.3)	9 (4.0)	30 [4.4]	24 (4.5)	79 (5.8)
17	7 (2.1)	0 (0.0)	0 (0.0)	7 (3.1)	14 [2.1]	14 (2.6)	55 (4.0)
18	5 (1.5)	0 (0.0)	0 (0.0)	5 (2.2)	10 [1.5]	10 (1.9)	66 (4.8)

**Table-IV:** Distribution of parasitic infections among Afghan children by age.

Intestinal parasites	Mean	SD	Median	p-value
Nematodes (-)	12.9	2.4	12.0	0.0001
Nematodes (+)	12.2	2.1	12.0	
Cestodes (-)	12.8	2.4	12.0	0.00003
Cestodes (+)	11.8	1.6	11.0	
Trematodes (-)	12.7	2.4	12.0	0.85
Trematodes (+)	12.4	1.6	12.0	
Protozoa (-)	12.8	2.4	12.0	0.0003
Protozoa (+)	12.2	2.1	12.0	
Total (-)	13.1	2.5	13.0	<0.000001
Total (+)	12.2	2.1	12.0	

## DISCUSSION

Afghanistan is considered to be a country with a high risk for developing a parasitic infections.^10^ Only 31% of all households have access to uncontaminated drinking water (26% of households in rural areas and 64% in urban areas). Contamination of water with pathogenic microorganisms is widespread and results in the increased occurrence of diarrheal diseases in Afghan population. Diarrheas are especially widespread among children and remain one of the main causes of morbidity. Only 5-7% Afghans have access to toilet facilities which meet sanitary standards.^11^,^12^

The Afghan healthcare is unable to satisfy the existing needs of the citizens because of a limited number of well-equipped healthcare facilities and shortages of qualified medical personnel. Owing to the fact that both the diagnostic and therapeutic capabilities of the Afghan healthcare are limited, it is frequently the case that Afghans are treated through trial and error (reports on morbidity rates are rarely confirmed by laboratory examination), and with a limited range of medications.^6^ Low public awareness of hygiene issues or the basics of disease prevention do not improve the situation. Therefore, the rates of parasitic infections in the Afghan community are exceptionally high. In February and March 2003 the United Nations World Food Programme (WFP), in cooperation with the World Health Organization (WHO) and the Afghan Ministries of Health and Education, conducted a cross-sectional parasitological examination in a group of 1001 children aged 8-15 from four Afghan provinces (Kabul, Nangarhar, Farah, Kandahar).^12^

The survey revealed the presence of intestinal parasites in 47.2% of the examined children. The most common pathogen was Ascaris lumbricoides, detected in 40.9% of the subjects. The authors of the survey suggested that regular administration of single-dose chemotherapy (albendazole 400 mg or mebendazole 500 mg) would be a cost-effective method of controlling soil-transmitted helminth infections at the public health level.^13^ In September 2004 WFP announced that the deworming campaign which had started six months before involved more than 4.5 million school children aged 6-12 in Afghanistan. The children were given a single oral dose of mebendazole.^14^ WHO recommends periodic administration of antihelminthic medicines as a public health intervention for children living in areas where the prevalence of soil-transmitted helminthiases is estimated at more than 20%.^15^

Unfortunately, the deworming campaign launched in the Third World countries has not been successful. In 2011 only 0.69% school-age children in the Eastern Mediterranean Region (including Afghanistan) received preventive antiparasitic therapy (albendazole or mebendazole).^15^ One of the reasons why such campaigns end in failure is unstable geopolitical situation in the region, as is the case with Afghanistan, where humanitarian aid is provided mainly by the international coalition forces stationing in the country since 2001. One of the participants in the International Security Assistance Force operation in Afghanistan was the Polish Military Contingent (PMC), which carried out its mandated tasks in Ghazni Province in eastern Afghanistan in the period 2008-2014. One of the components of the PMC was a medical unit whose task was to promote disease prevention and provide treatment to the local population. In March 2013 medical personnel from Polish Military Contingent in Afghanistan designed and implemented the project Capacity building of health care system in Ghazni Province (financed by the Polish Ministry of National Defence).

One of the aims of this project was to purchase thousands of medicine doses (albendazole, metronidazole, praziquantel) for the infected children treated in the Ghazni Provincial Hospital, as well as for the infected students attending high schools^16^ in the capital of Ghazni Province. Owing to high prevalence of multiple infections (nematodes, cestodes, trematodes, protozoa) in the Afghan community, it seems that a mass deworming campaign with single-dose chemotherapy (albendazole 400 mg or mebendazole 500 mg) may prove ineffective in eradicating intestinal parasites in the local population. The effectiveness of deworming may be enhanced by increasing the dosage of albendazole (400 mg for three-five days)^17^,^18^ or mebendazole (600 mg for three days),19 which, considering the low cost of the medication, seems an acceptable form of the antiparasitic therapy.

## CONCLUSIONS

The Afghan community is an example of population characterized by a high rate of parasitic infections. Owing to high prevalence of multiple infections among people living in Afghanistan, it seems that a mass deworming campaign with a single-dose chemotherapy may prove ineffective in eradicating intestinal parasites in the local population.
